# Wildfire-Induced CO Plume Observations From NAST-I During the FIREX-AQ Field Campaign

**DOI:** 10.1109/jstars.2021.3059855

**Published:** 2021-02-19

**Authors:** Daniel K. Zhou, Allen M. Larar, Xu Liu, Anna M. Noe, Glenn S. Diskin, Amber J. Soja, G. Thomas Arnold, Matthew J. McGill

**Affiliations:** NASA Langley Research Center, Hampton, VA 23681 USA; NASA Langley Research Center, Hampton, VA 23681 USA; NASA Langley Research Center, Hampton, VA 23681 USA; NASA Langley Research Center, Hampton, VA 23681 USA; NASA Langley Research Center, Hampton, VA 23681 USA; NASA Langley Research Center, Hampton, VA 23681 USA, and also with the National Institute of Aerospace, Hampton, VA 23666 USA; NASA Goddard Space Flight Center, Greenbelt, MD 20771 USA, and also with Science Systems and Applications, Inc., Lanham, MD 20706 USA; NASA Goddard Space Flight Center, Greenbelt, MD 20771 USA

**Keywords:** Air quality, carbon, fires, infrared measurements, remote sensing

## Abstract

The fire influence on regional to global environments and air quality (FIREX-AQ) field campaign was conducted during August 2019 to investigate the impact of wildfire and biomass smoke on air quality and weather in the continental United States. One of the campaign’s scientific objectives was to estimate the composition of emissions from wildfires. Ultraspectrally resolved infrared radiance measurements from aircraft and/or satellite observations contain information on tropospheric carbon monoxide (CO) as well as other trace species present in fire emissions. A methodology for retrieving tropospheric CO from such remotely sensed spectral data has been developed for the National Airborne Sounder Testbed-Interferometer (NAST-I) and is applied herein. Retrievals based on NAST-I measurements are used to demonstrate CO retrieval capability and characterize fire emissions. NAST-I remotely sensed CO from ER-2 flights are evaluated with concurrent *in situ* measurements from the differential absorption carbon monoxide measurements flown on the NASA DC-8 aircraft. Enhanced CO emissions along with plume evolution and transport from the fire ground site locations were captured by moderate vertical and high horizontal resolution observations obtained from the NAST-I IR spectrometer; these were intercompared and verified by the cloud physics lidar and the enhanced MODIS airborne simulator also hosted on the NASA ER-2 aircraft. This study will be beneficial to the science community for studying wildfire-related topics and understanding similar remotely sensed observations from satellites, along with helping to address the broader FIREX-AQ experiment objectives of investigating the impact of fires on air quality and climate.

## I. Introduction

CHEMISTRY and composition of smoke from wildfires and agricultural burning are studied to improve our understanding of the relationship between combustion burning and air quality, weather, and climate forecasts [1]. The fire influence on regional to global environments and air quality (FIREX-AQ) of August 2019 is the first joint field campaign conducted by NOAA and NASA addressing wildfire emissions and their impact on air quality and climate. It is dedicated to the sampling and characterization of fires and their impact on air quality and weather from the point of trace species emissions [2], [3]. Carbon monoxide (CO) is one of the major pollutants due to combustion; air particles downwind of combustion often show elevated CO resulting from photochemical production [4]. The significance of CO in atmospheric chemistry was recognized long ago when a photochemically driven chain reaction was recognized linking the tropospheric cycles of CO, methane (CH_4_), and ozone (O_3_) with those of the hydroxyl radical (OH) and hydroperoxyl radical (HO_2_) [5]. Tropospheric chemical reactions involving CO extend their influence on air quality and the global climate through accumulation of greenhouse gases. Due to its relatively long lifetime (averaging about 2 months in the atmosphere), CO can be transported a great distance from its original source.

The critical role of satellite observations has been established by providing necessary global and regional observations in order to understand the complex chemistry and transport processes involved in regional air pollution chemistry and its influence on the global environment. In December 1999, the measurement of pollution in the troposphere (MOPITT) instrument was launched aboard the TERRA satellite [6], [7] for space-based measurement of CO and CH_4_. In July 2004, the tropospheric emission spectrometer (TES) instrument was launched aboard the Aura satellite to detect tropospheric trace species. One of the objectives of these missions was to monitor global CO distribution [8]. Current ultraspectral infrared sounders on a series of weather satellites, i.e., the atmospheric infrared sounder (AIRS) on Aqua [9], the interferometer atmospheric sounding instrument (IASI) on MetOp [10], and the cross-track infrared sounder (CrIS) on SNPP and JPSS [11] also have the ability to observe trace gases including CO. However, remotely sensed CO from these weather satellites has a lower vertical and horizontal resolution due to the satellite sensor spectral resolutions and spatial footprint sizes, i.e., 12–15 km field-of-view (FOV) or 45 km field-of-regard (FOR).

Similar ultraspectral infrared sounders flown on high-altitude aircraft can provide such measurements with a higher spectral resolution and much smaller footprint size. The National Airborne Sounder Testbed-Interferometer (NAST-I) has been successfully operating on high-altitude aircraft since 1998 [12]-[14]. NAST-I onboard NASA high-altitude research aircraft serves as a spaceborne instrument simulator. NAST-I provides high-spatial linear resolution equal to 13% of the aircraft altitude at nadir (2.6 km FOV on the ground from an ER-2 altitude of 20 km) and 13 FOVs across the aircraft track from 13 NAST-I scan angles (~3.4 km apart on the ground from an ER-2 altitude of 20 km). NAST-I spatially scans and provides high-spectral resolution (0.25 cm^-1^) measurements within the spectral region of 645–2700 cm^-1^. It serves as an ideal validation sensor since it measures the same Level-1 data product as many of the sensors it helps to validate (i.e., infrared spectral radiance) and does so at higher spectral and spatial resolutions [15]. NASA Langley Research Center analysis is further benefited by implementing a set of internal algorithms to enable an independent assessment of derived Level-2 products [16]. Airborne field campaigns are generally designed to allow retrieval algorithm enhancements and validation with a set of rich coincidental measurements from other sensors [17]–[19].

In this communication, we demonstrate the ability of NAST-I to monitor tropospheric CO distributions under the extreme concentration conditions associated with wildfires. Different from other airborne field experiments that NAST-I has participated in, FIREX-AQ allowed the aircraft sensors to observe the very unique environment within and surrounding wildfire combustion. NAST-I was part of the scientific payload on board the NASA ER-2 aircraft during FIREX-AQ; it provided the opportunity to observe polluted regions and collect ultraspectral radiance data for demonstrating the NAST-I ability to retrieve elevated trace species (e.g., CO) concentration amounts induced by wildfire combustion. The objectives of this article are to use the data collected during FIREX-AQ to demonstrate NAST-I CO retrieval capability, to intercompare and evaluate NAST-I remotely sensed CO with the differential absorption carbon monoxide measurement (DACOM) *in situ* CO measurements [20], to assess plume correlation between CO and smoke-dust detected by cloud physics lidar (CPL) [21] and the enhanced MODIS airborne simulator (eMAS) [22], and to examine the relationship between the total carbon emission and elevated atmospheric CO amount during fire progression and plume evolution. All evidence leads to the conclusion that an elevated CO plume near a wildfire location was indeed produced by the wildfire combustion and subsequent advection; its evolution and transport were captured by NAST-I measurements during FIREX-AQ. NAST-I data presented in this article were collected under clear-sky conditions. NAST-I CO profile cannot be retrieved under opaque clouds as infrared measurements are not able to penetrate opaque clouds [14]. NAST-I remotely sensed CO profile observations are relevant, and they can contribute to applications such as environment and/or air quality (pollution), atmospheric chemistry and dynamics, and climate monitoring and studies.

The remainder of the article is organized as follows. A brief description of the NAST-I retrieval algorithm and CO retrieval results will be given in Section II. NAST-I CO retrieval intercomparisons and evaluation with DACOM *in situ* CO measurements from the NASA DC-8 aircraft are presented in Section III. Additionally, the CO plume assessments with CPL and eMAS observations from the NASA ER-2 aircraft along with total carbon emissions from the ground are presented. Conclusions follow in Section IV.

## II. CO Retrieval and Its Plume

**Fig. 1.**
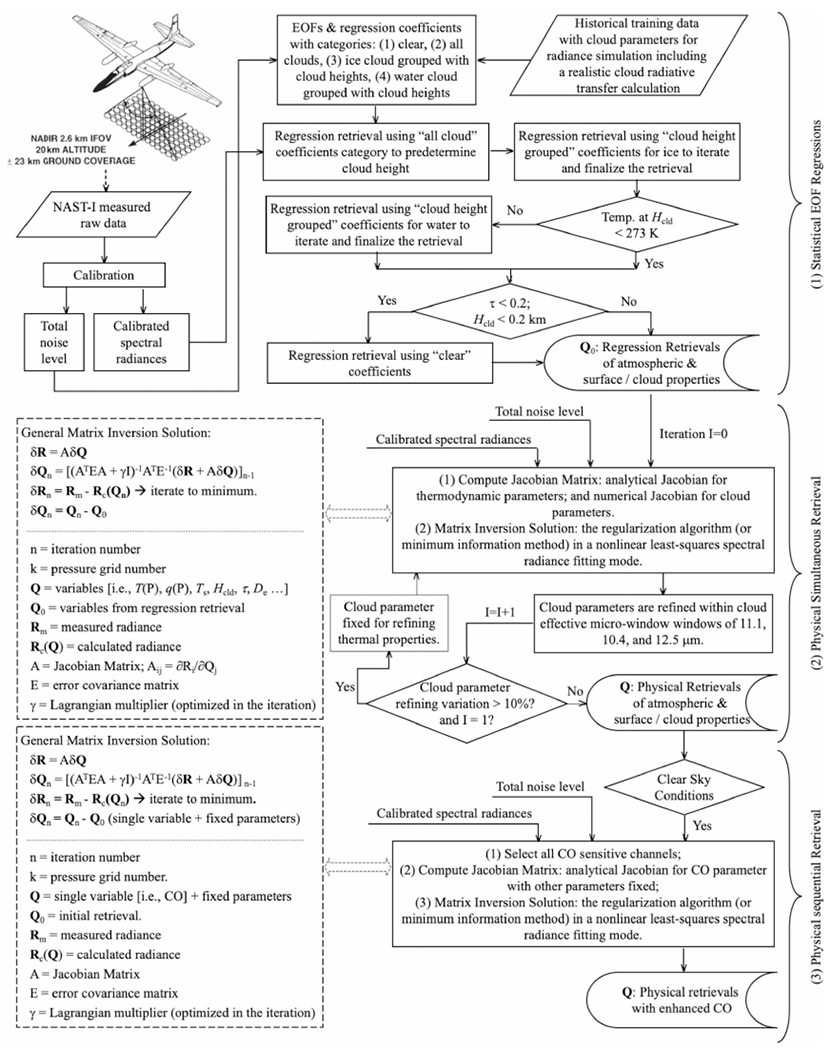
NAST-I CO retrieval algorithm flowchart, where *P* is pressure, *T(P)* is temperature at *P, q(P)* is water mixing ratio at *P, T*_*s*_ is surface temperature, *τ* is cloud optical depth, *D*_*e*_ is cloud particle size, and *H*_cld_ is cloud top height [14], [27]–[29].

The intent of the measurement of chemical abundance (such as tropospheric CO) is for monitoring air quality and the initialization of climate process models. Several existing inversion algorithms for retrieving CO from satellite remote sensing measurements in the infrared have been summarized [23]. CO amounts retrieved from satellite thermal infrared data from AIRS, IASI, and CrIS are found elsewhere [24]-[26]. The NAST-I retrieval algorithm was developed, tested, and evaluated mainly for atmospheric temperature and moisture profiles, surface skin temperature and spectral emissivity under cloud-free conditions, or cloud microphysical parameters in cloudy conditions [14], [27]. In the early years of the NAST-I program, field campaigns and algorithm development for retrieving these thermodynamic parameters were mainly for weather satellite sensor applications and associated algorithm development, risk mitigation, and validation [17]-[19]. The NAST-I CO retrieval algorithm was also developed [28] and later improved by the implementation of a surface emissivity retrieval [29]. To summarize the NAST-I retrieval algorithm, Fig. 1 presents a CO retrieval algorithm flowchart showing the three steps of: 1) statistical empirical orthogonal function (EOF) regression; 2) physical simultaneous retrieval; and 3) physical sequential retrieval for CO to further minimize the error and increase vertical sharpening for CO profiles.

**Fig. 2.**
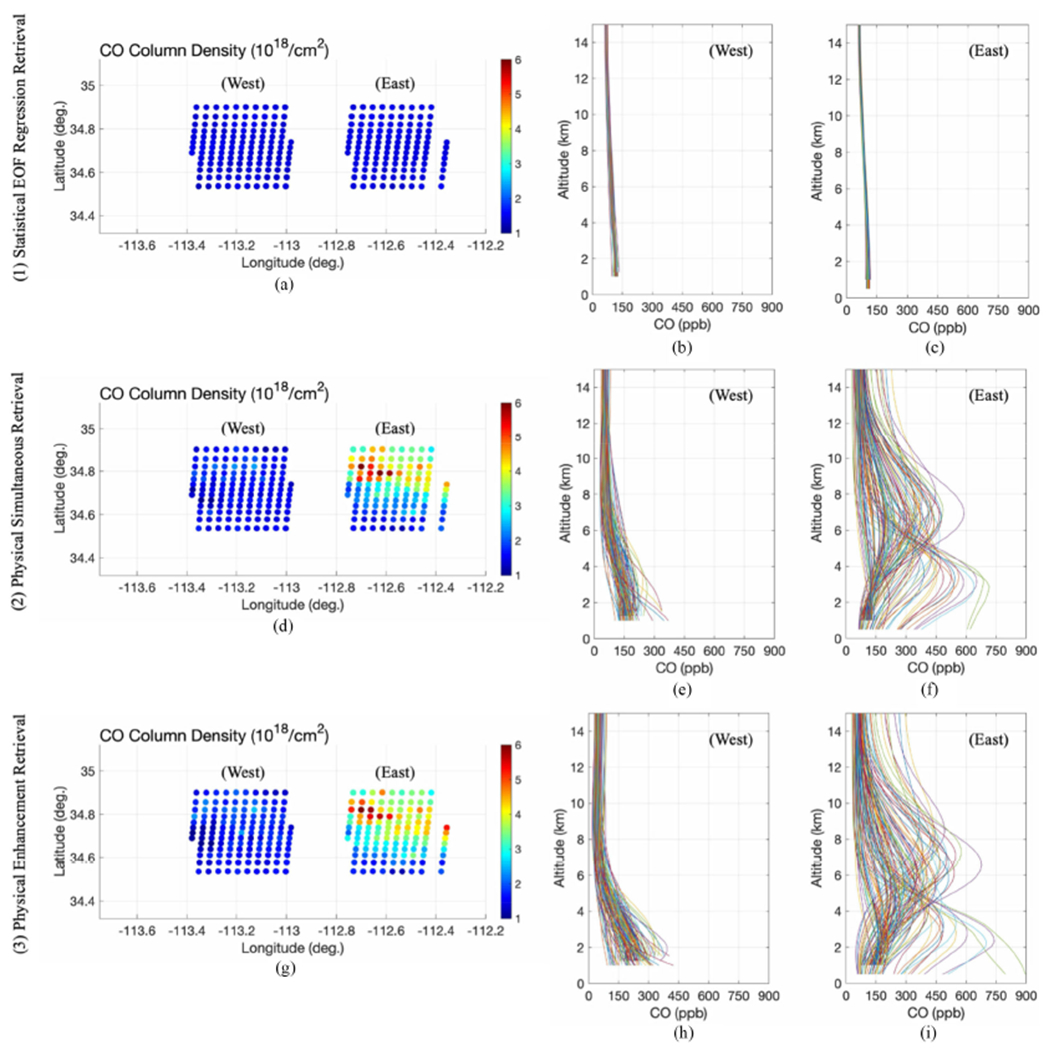
NAST-I CO retrieval progression through statistical EOF regression (top panel), physical simultaneous retrieval (middle panel), and physical sequential retrieval (bottom panel) from relatively clean (upwind, or west) and polluted (downwind, or east) regions near Sheridan fire on August 21, 2019 (see text).

We have selected one of the ER-2 flight sorties from August 21, 2019 over the Sheridan wildfire to demonstrate the NAST-I CO retrieval using the step-by-step algorithm described in Fig. 1. The Sheridan wildfire was caused by lightning, started on August 5, 2019, and located at approximately 37 km (23 miles) northwest of Prescott, Arizona in the Chino Valley District (34.80° latitude, -112.85° longitude). As the ER-2 flew over the Sheridan fire location area, the wind was blowing from west to east and the smoke-dust plume was evolving and transporting downstream with the wind from the fire location. Two groups of NAST-I measurements, one from west of the Sheridan fire and the other from the east, are used to demonstrate NAST-I CO retrievals from a relatively clean upwind area (west of the fire) and a polluted downwind area (east of the fire). Fig. 2 plots the CO retrievals from statistical EOF regressions (top panel), physical simultaneous retrievals (middle panel), and physical sequential retrievals (bottom panel). Plots on the left are CO column density; in the middle, CO profiles from a relatively clean area (west of the fire); and on the right, CO profiles from a polluted area (east of the fire). The data plotted in the figure show that enhanced CO physical sequential retrieval is helping to further reduce the noise and increase the profile vertical sharpening.

**Fig. 3.**
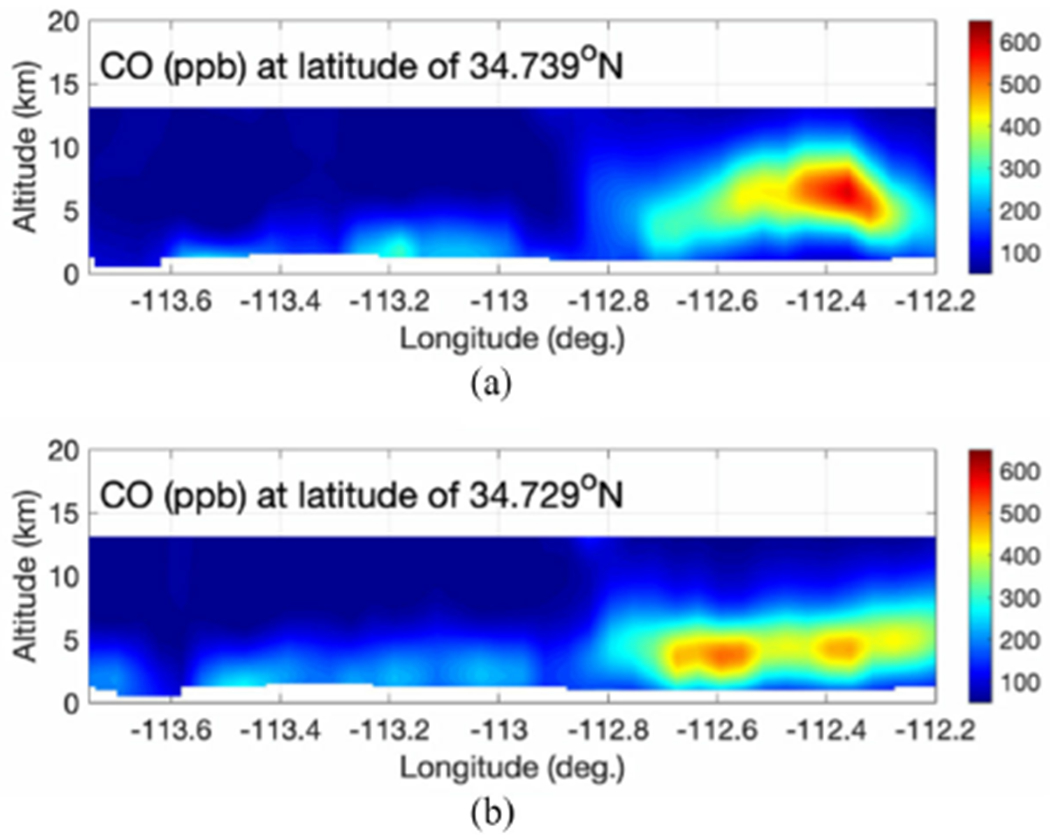
CO time-evolution shown in its vertical profile cross sections upwind and downwind of the Sheridan fire location (about 140 km) from (a) 23:28:13–23:39:37 UTC of August 21, 2019 to (b) 00:38:35–00:49:24 UTC of August 22, 2019. (a) and (b) are about 70 min apart.

**Fig. 4.**
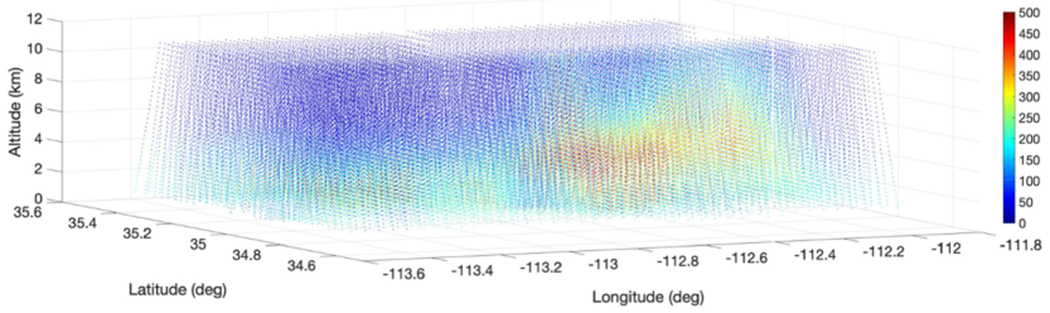
NAST-I 3-D CO (ppb) distribution shows the plume evolution and transport near the Sheridan fire ground location (34.80° latitude, −112.85° longitude) from August 21, 2019.

The NASA ER-2 aircraft was flown over the Sheridan wildfire location back and forth five times in west to east and east to west from August 21 23:26:45 UTC to August 22 00:52:00 UTC to capture the CO plume and its time-evolution. Fig. 3 plots the cross sections of the CO vertical profiles near the Sheridan wildfire (about 140 km). Two overpasses are about 70 min apart over the same area showing CO plume evolution and downwind transport from the fire location. Unlike nominal free tropospheric CO, the fire-induced CO plume varies rapidly in space and time, depending on the combination of the specific fire (e.g., fire fuel types) and weather conditions (e.g., wind). Fig. 4 illustrates the 3-D field distribution of CO as observed by NAST-I from the ER-2 flight sortie of August 21, 2019. From these particular observations, the CO plume went as high as 10–12 km in the atmosphere as we compare CO profiles between upwind nominal tropospheric CO background and the downwind fire-induced CO plume. A high-intensity CO plume was observed at least 70 km downwind (east of the fire location) and ~50 km cross wind (i.e., north-south direction). The plume was moving to the area further east of the ER-2 flight region (also shown in Fig. 3). Significant differences in tropospheric CO distributions between upwind and downwind areas are associated with the plume evolution and transport. Small inconsistencies at overlapped geolocations are from time-evolution observed by the ER-2 aircraft being flown back and forth five times, as shown, for example, in Fig. 3. Nevertheless, Fig. 4 gives an overall view of the Sheridan wildfire-induced CO plume in the area captured by NAST-I measurements under biomass burning and weather conditions.

## III. Evaluation and Discussion

The FIREX-AQ field campaign collected a wealth of coincidental data from numerous remote and *in situ* sensors across multiple aircraft, ground, and satellite observational platforms. Measurements from sensors on aircraft, ground, and satellite are valuable for CO evaluation and science investigation to help characterize different spatial and temporal scales of the plume and associated variability. In this communication, we present a NAST-I CO retrieval intercomparison and evaluation with DACOM CO *in situ* measurements from the NASA DC-8 aircraft. Moreover, the plume intensity, evolution, and transport are assessed with the smoke-aerosol plume as observed by the CPL and the eMAS from the same ER-2 aircraft. Finally, fire-induced CO amounts in the stratosphere were used to show fire progression linked to total carbon emissions caused by fire combustion.

CO remotely sensed by NAST-I from the ER-2 aircraft can be evaluated with DACOM CO *in situ* measurements from NASA DC-8 aircraft. A few DC-8 sorties were spatially coincident with the ER-2 sorties at the same fire locations but, in general, they had time-lags of a few hours. There was one exception wherein both spatial and temporal coincidence was achieved between the two aircrafts, specifically, on August 6, 2019 over the Williams Flats fire that was located at about 11 km (7 miles) southeast of Keller, Washington (47.95° latitude, -118.65° longitude). Fire-induced CO changes rapidly with the nature of the wildfire depending on the fire fuel and weather conditions. Thus, for evaluation purposes, datasets used for intercomparison must be close enough in terms of location and time. It is worth mentioning that *in situ* and remotely sensed observations look at very different parts of the atmosphere, so it is impossible to find a perfect spatial and temporal data match for intercomparison. For this reason, remotely sensed CO from NAST-I and *in situ* CO from DACOM can appear like very different measurements.

**Fig. 5.**
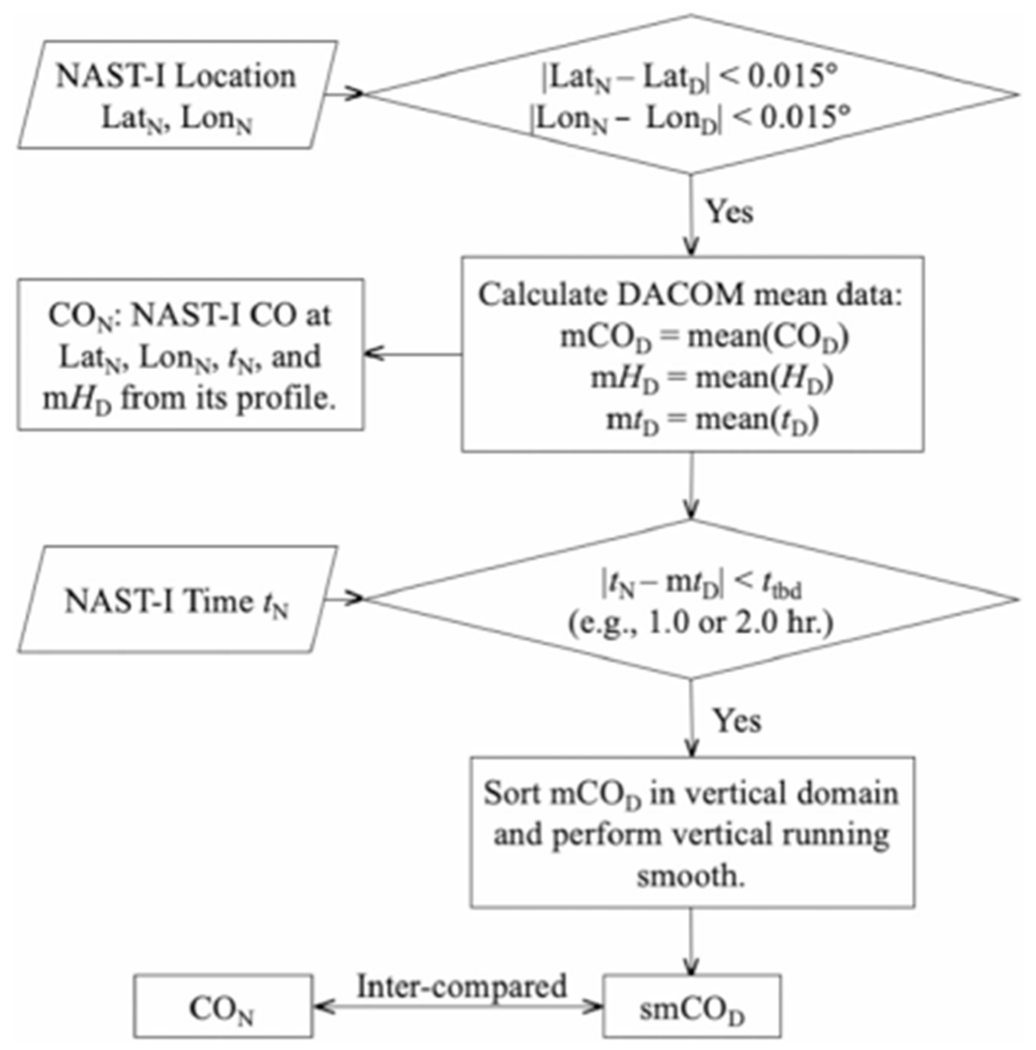
Flowchart for DACOM *in situ* CO measurement degradation to NAST-I spatial–temporal scales (see text), where *H* is height and *t* is time. Subscripts D and N are for DACOM and NAST-I, respectively.

**Fig. 6.**
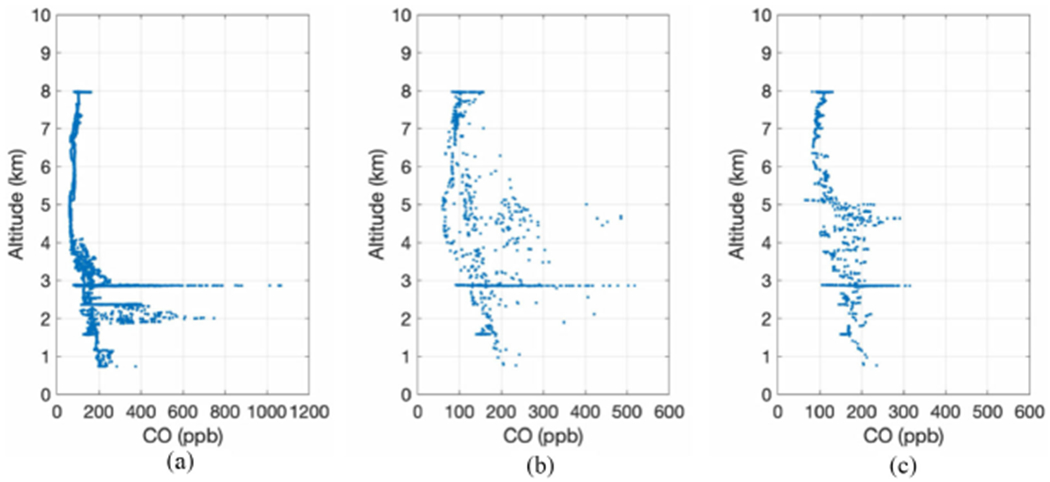
DACOM *in situ* CO degradation step-by-step to counterpart NAST-I data having Δlatitude and Δlongitude within ±0.015°, and ΔUTC within ±2 h: (a) CO_D_; (b) mCO_D_; and (c) smCO_D_ as described in Fig. 5 flowchart.

**Fig. 7.**
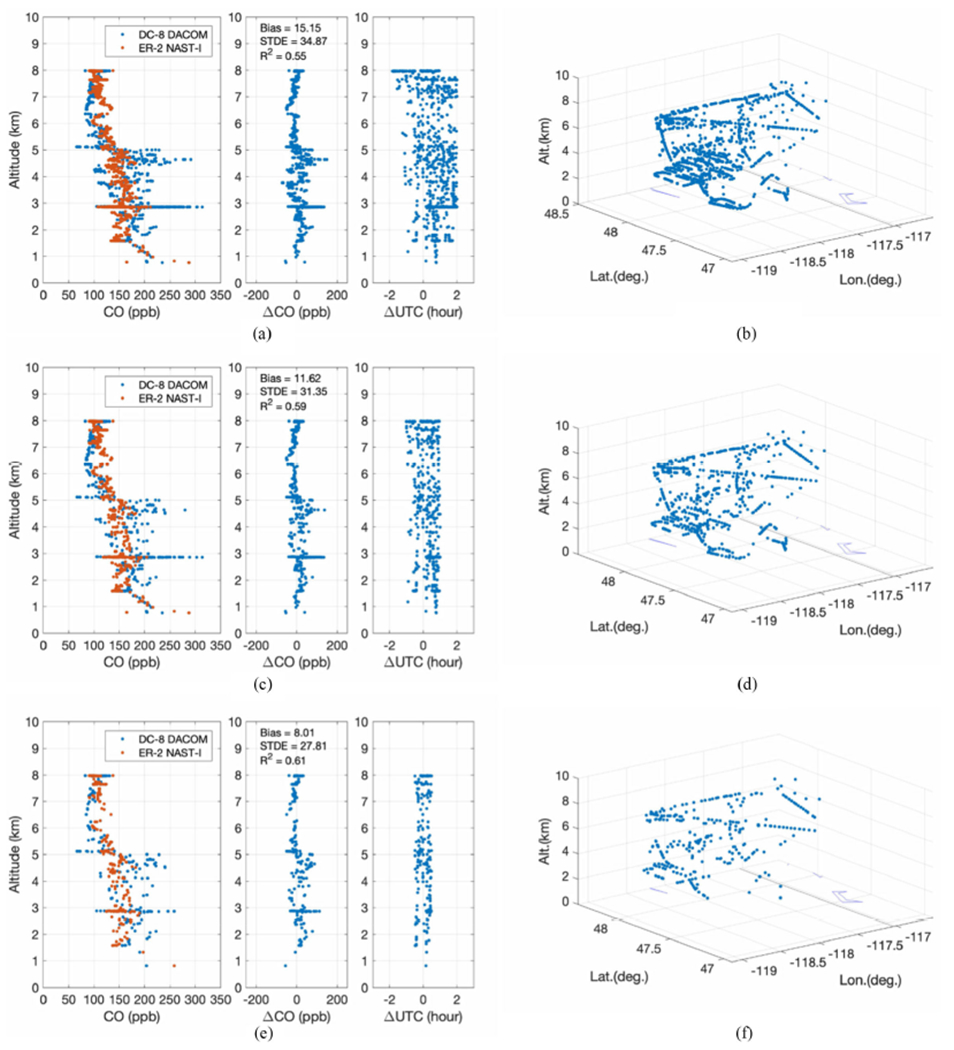
Intercomparison between DACOM (smCO_D_) and NAST-I (CO_N_) from August 6, 2019: (a) smCO_D_ and CO_N_ comparison with statistical parameters of bias, STDE, and *R*^2^, and associated ΔUTC is less than ±2 h and (b) data locations (near Williams Flats fire). (c) and (d) are the same as (a) and (b) but with associated ΔUTC less than ±1 h; and (e) and (f) are the same as (a) and (b) but with associated ΔUTC less than ±0.5 h.

Fig. 5 is a flowchart showing how DACOM data are degraded in spatial domain to best match NAST-I spatial resolution within a specific time difference (ΔUTC) of two measurements. This is the methodology currently used in the comparisons presented herein. The quasi-matched DACOM and NAST-I data have Δlatitude and Δlongitude within ±0.015° (~3 × 3 km) and ΔUTC within ±2 h (or a different ΔUTC window). At the same time, DACOM mean data (mCO_D_) are also averaged vertically in altitude dimension. After additional vertical smoothing (i.e., a running average), smCO_D_ are the vertically smoothed mean within a cube equivalent to NAST-I CO resolution, assuming DACOM data samples collected in the cube can represent the mean of the cube. In reality though, *in situ* data points within the area (or cube) may not be a good representation of what was measured by the remote sensor, especially in a highly nonuniform area such as a wildfire smoke plume. The criteria (i.e., Δlatitude, Δlongitude, and vertical smooth factor) for averaged smCO_D_ to counterpart NAST-I CO data (CO_N_) can be adjusted to meet the best fitting results between smCO_D_ and CO_N_, where subscripts D and N are for DACOM and NAST-I, respectively. Following the flowchart in Fig. 5, Fig. 6 shows step-by-step averaging of DACOM data to the counterpart NAST-I data. DACOM CO data from Fig. 6(c) (i.e., smCO_D_) is then used to compare with CO_N_. The methodology for degrading DACOM CO to NAST-I alike is straightforward. The outcome illustrates large differences between CO_D_ [see Fig. 6(a)] and smCO_D_ [see Fig. 6(c)]. This shows that DACOM only samples a small part of the extremely heterogeneous environment over which NAST-I observes its FOV mean. Fig. 7(a) [or7(c), 7(e)] and 7(b) [or7(d), 7(f)] plot the DACOM and NAST-I data used for intercomparison and where these data are located. Multiple data points at the same altitude are from different locations and/or time. Profiles are not from a single location and time, rather these data points are from the area shown in Fig. 7(b) [or 7(d), 7(f)] during the DC-8 and ER-2 aircraft overlapping period from approximately 18:00 to 23:45 UTC, August 6, 2019. ΔUTC between DACOM and NAST-I for each single data point is less than ±2, ±1, and ±0.5 h for the upper [see Fig. 7(a) and 7(b)], middle panel [see Fig. 7(c) and 7(d)], and lower panel [see Fig. 7(e) and 7(f)], respectively. The agreement is better with a smaller ΔUTC in terms of the mean bias, standard deviation of difference (STDE), and coefficient of determination R^2^. A positive result is shown in Fig. 7 despite the nature of heavier rapid variation from the wildfire-induced CO plume and the difference between *in situ* and remotely sensed measurements.

**Fig. 8.**
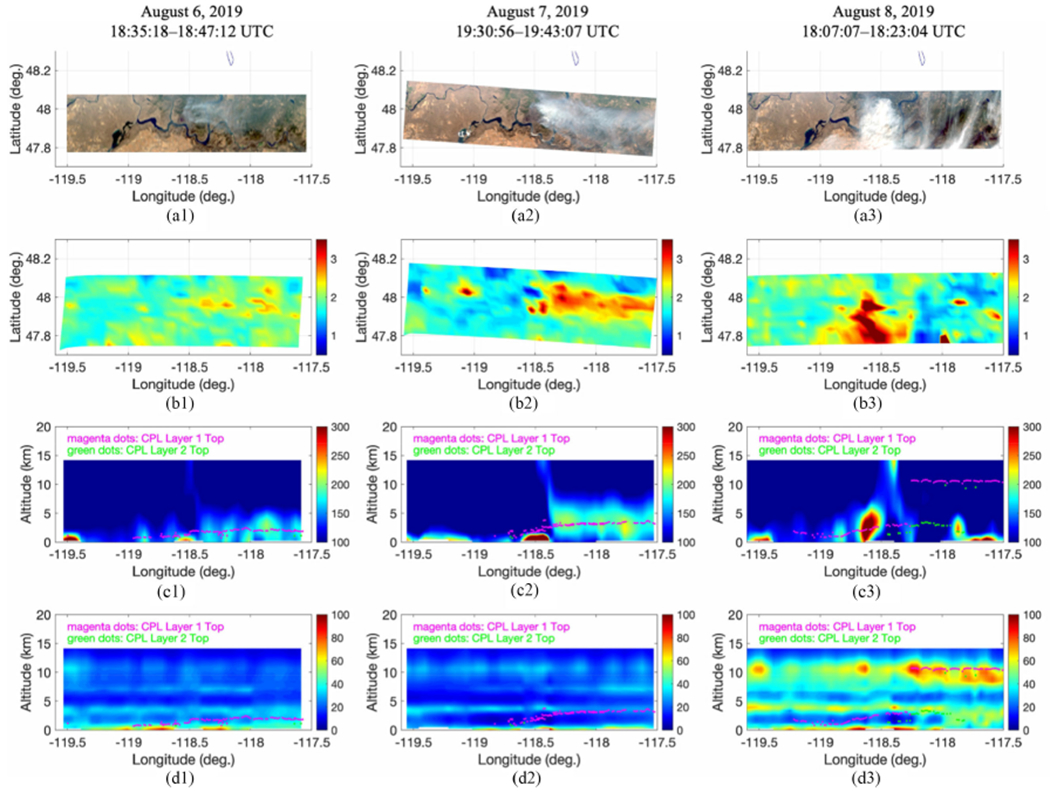
Williams Flats fire progression from August 6 (left column) to August 7 (middle column), then to August 8 (right column): (a) eMAS true-color imageries; (b) CO column density (10^18^/cm^2^); (c) CO vertical profile (ppb) cross section with CPL layer top; and (d) relative humidity vertical profile (%) cross section with CPL layer top (see text).

The ER-2 aircraft flew to the Williams Flats wildfire location for three consecutive days to observe fire progression. The CO plume intensity, evolution, and transport associated with the fire-produced smoke can be verified by the observations of eMAS and CPL from the same aircraft. The aircraft flew between longitude 117.5°W and 119.6°W with a near constant latitude of 47.9°N, covering 160 km in distance, as shown in Fig. 8. The measurements from both eMAS and CPL confirmed that elevated CO observed by NAST-I is representative and indeed produced by the wildfire. The eMAS is an airborne scanning spectrometer that acquires high spatial resolution (50 m) imagery of cloud (or smoke-aerosol) and surface features. The eMAS has a swath width of about 37 km from the ER-2 flight altitude of 20 km. eMAS imagery from wildfires and its induced smoke-aerosol are observed to identify fire intensity and smoke-aerosol evolution. The Williams Flats fire progression is shown in eMAS measurements. Fig. 8(a) (the top panel) plots eMAS true-color imagery showing the smoke-aerosol increased from August 6 through 8, 2019, as the measurements taken from ER-2 indicate. It shows the eastward downwind transport during August 6 and 7 before it switched to southern downwind transport on August 8. The NAST-I CO column density and CO nadir vertical profile cross sections are shown in Fig. 8(b) and 8(c), respectively. The CO intensity increased in the downwind transport direction, which is consistent with eMAS smoke-aerosol day-to-day observations.

Smoke-aerosol layers are also measured by CPL. The CPL is a backscatter lidar designed to provide multiwavelength measurements of cirrus, subvisual cirrus, and aerosols with high temporal and spatial resolution (i.e., ~200 m in horizontal) along the flight track of the ER-2 aircraft. The CPL-observed cloud and/or aerosol layer top height is plotted on the NAST-I CO vertical profile cross section in Fig. 8(c). The CO plume downwind of the fire location is shown to be correlated with CPL smoke aerosol layer, while the upwind CO is considered as the atmospheric CO nominal background. It can be further identified whether the CPL layer is from aerosol or cloud by the relative humidity retrieved from NAST-I measurements [see Fig. 8(d)]. Relative humidity was low for both August 6 and 7, indicating that there were no clouds present. Therefore, we conclude that the CPL measured layer was an aerosol layer produced by the Williams Flats fire. From August 8, however, the CPL layer top at ~11 km was from cirrus clouds where NAST-I-retrieved relative humidity was about 90%, while a lower layer was from fire-induced smoke-aerosols. It is worth mentioning that CPL has a much higher resolution (200 m) than that of NAST-I (2.6 km). Fire-induced smoke-aerosol is observed to identify smoke-aerosol layer and evolution. Aerosol and CO distributions could vary; and their relationship could be exceedingly complex, even though they are both induced by the same wildfire. It is not studied quantitatively in the scope of this work. However, the correlation of CO and smoke-aerosol plumes induced by the Williams Flats wildfire is recognized. Similar results showing the correlation between wildfire produced CO plumes (observed by NAST-I) and smoke-aerosol plumes (observed by CPL and eMAS) are found in other FIREX-AQ cases such as the Sheridan fire-induced CO plume discussed in Section II.

**Fig. 9.**
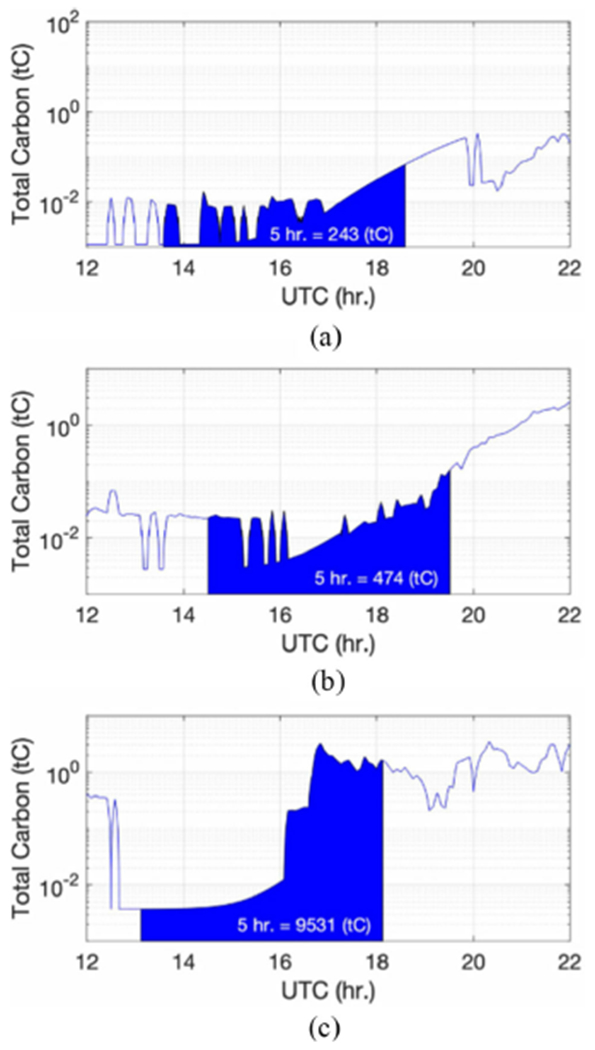
Total carbon emissions from the Williams Flats fire for (a) August 6, (b) August 7, and (c) August 8, 2019. The area experienced a 5-h total carbon emission prior to the ER-2 observations shown in Fig. 8.

Another perspective on fire variation can be obtained by examining the total carbon emission from the fire site. Wildfire-induced CO in the troposphere should be linked or proportional to the carbon emission from the fire. Carbon emission from biomass burning can be estimated by a methodology described elsewhere [30]-[32]. Analyses on the carbon emission have been performed for FIREX-AQ including the Williams Flats fire. The total carbon emitted from the Williams Flats fire as a function of time is plotted in Fig. 9. The total carbon amounts from t — 5 to t h (where t is ER-2 UTC time shown in Fig. 8) are estimated to be approximately 243, 474, and 9531 tC, from August 6–8, respectively. We assume that fire-induced CO in the troposphere cumulated in (or moving in and out of) NAST-I observation space is associated with the carbon emissions from the ground fire during the previous 5 h. This is an irregular assumption as it depends on the weather (i.e., wind), but it should be satisfactory as we are looking at a relative quantity of ground carbon emission versus tropospheric CO amount. The estimation of total carbon emission from these three consecutive days (August 6–8, 2019) shows the Williams Flats fire progression. Tropospheric CO observed by NAST-I shown in Fig. 8(b) and 8(c) is related to the total carbon emission from the Williams Flats fire, reflecting their positive correlation. Tropospheric elevated CO increases as ground total carbon emission increases, which is observed from these three consecutive days. Hence, together with eMAS and CLP observations, we attribute the NAST-I observed elevated tropospheric CO to be fire induced.

## IV. Conclusion

The FIREX-AQ field campaign with multiple aircraft *in situ* and remotely sensed observations provides the characterization of distributions of chemical species, such as CO, induced by wildfires. This unique dataset is very much desirable in validating CO retrieval algorithms and results with an elevated CO amount. Two wildfire cases from the FIREX-AQ experiment dataset are reported herein, one is from the Sheridan fire and the other is from the Williams Flats fire. Several major summary items and conclusions can be obtained from this work.

1. Wildfire-induced CO plumes in the troposphere, in conjunction with their evolution and transport, are readily identified with NAST-I measurements.
2. NAST-I retrieval ability is demonstrated showing the contrast between nominal atmospheric background levels and fire-induced elevated CO profiles.
3. NAST-I remotely sensed CO is evaluated by favorable intercomparisons with the DACOM *in situ* CO measurements, which show a positive agreement.
4. Plume characterization correlation between CO and smoke-dust detected by the CPL and eMAS is assessed and presented a good correspondence.
5. Elevated tropospheric CO induced by the wildfire is associated and correlated with the total carbon emission from biomass burning.

First-of-a-kind wildfire-induced CO plume measurements obtained by the NAST-I ultraspectral remote sensor on board the ER-2 suborbital aircraft have shown the intensity and size of wildfire plumes in a high spatial resolution of 2.6 km. Remotely sensed CO from NAST-I is different from *in situ* measured CO as they observe different spatial–temporal parts of the atmosphere, but remotely sensed measurements do have the advantage of giving broader spatial and temporal context by rapidly covering a large field of observation, as shown in Fig. 4. NAST-I onboard the ER-2 suborbital aircraft functions as a spaceborne instrument emulator, demonstrating the ability to monitor CO by an ultraspectral infrared sounder from space with a higher spatial resolution. Data collected by current satellite sounders such as AIRS, CrIS, and IASI can be further investigated in conjunction with FIREX-AQ datasets to better understand CO retrieval sensitivity and/or ability (e.g., vertical resolution) due to varying instrumental aspects such as FOV size, spectral coverage, spectral resolution, and noise performance. Also, interest in the relationship between CO plume (intensity and size) and total carbon emission from ground biomass burning, and smoke-aerosol distribution from eMAS and CPL measurements, stems in part from the availability of the data and analysis from the FIREX-AQ experiment which will promote further investigation.

NAST-I was successfully operated during all ER-2 flights of the FIREX-AQ experiment (a total of 11 flights and 50+ h of science data collected). NAST-I retrievals (e.g., atmospheric temperature, relative humidity, and CO profiles, also surface skin temperature and CO column density), together with experiment data from other satellite/aircraft/ground measurements and analysis from the FIREX-AQ campaign are available [33] for the science community to study wildfire-related topics as described by the overarching objective of FIREX-AQ experiment [2] and beyond.
